# Mixed Trabecular and Mucinous Carcinoid Arising in the Background of a Mature Ovarian Cystic Teratoma

**DOI:** 10.7759/cureus.77013

**Published:** 2025-01-06

**Authors:** Syed Muhammad Hammad Ali, Haseeb Ahmed Khan, Asim Malik, Sana Ali, Ahmad Hussain

**Affiliations:** 1 Surgery, Fatima Memorial Hospital College of Medicine & Dentistry, Lahore, PAK; 2 Pathology, Fatima Memorial Hospital College of Medicine & Dentistry, Lahore, PAK; 3 Obstetrics and Gynecology, James Cook University Hospital, Middlesbrough, GBR; 4 General Surgery, King Edward Medical University, Lahore, PAK

**Keywords:** carcinoid tumor, dermoid cyst, neuroendocrine tumors, ovarian neoplasms, teratoma

## Abstract

Ovarian carcinoid tumors, originating from mature ovarian cystic teratomas (dermoid cysts), are rare neuroendocrine tumors. The synchronous occurrence of trabecular and mucinous carcinoid tumors within a single ovarian teratoma is infrequent and has limited documentation. We present the case of a 67-year-old postmenopausal woman presenting with abdominal pain, constipation, and urinary retention, but without features of carcinoid syndrome. Ultrasonography identified a complex, multiloculated cystic mass in the right ovary. Laboratory assessments, including CA-125 levels, were unremarkable. The cyst was aspirated and surgically excised, with the patient experiencing an uncomplicated recovery. Histopathological and immunohistochemical analyses confirmed a mixed trabecular and mucinous carcinoid tumor within the teratoma. A six-month follow-up ultrasound revealed no recurrence, and the patient remained symptom-free. This case highlights the importance of considering synchronous, asymptomatic carcinoid tumors in mature ovarian teratomas during gross and histopathological examinations. Additionally, the presence of a mucinous carcinoid should warrant a high suspicion of metastasis and subsequent recurrence. Further research is needed to establish management and recurrence monitoring guidelines.

## Introduction

A mature cystic teratoma, commonly referred to as a dermoid cyst, is the most prevalent benign ovarian germ cell tumor in the female reproductive system. It is characterized by its composition of tissues that may originate from all three germ cell layers: the endoderm, mesoderm, and ectoderm [1]. In contrast, primary ovarian carcinoid tumors are exceptionally rare, constituting approximately 0.1% of all ovarian neoplasms [2,3]. These tumors can independently arise within teratomas and are typically benign. Ovarian carcinoids are categorized into various subtypes, including insular, strumal, trabecular, mucinous, and mixed forms. Among these subtypes, mucinous and trabecular carcinoids are particularly uncommon [4].

Peri- or post-menopausal women, with a median age of 53 years, are most commonly affected by these tumors [5]. They commonly manifest as pelvic masses, symptoms resulting from the compression of nearby structures, and abdominal pain. However, features of carcinoid syndrome are rarely observed [6].

In this report, we present the case of a multiparous postmenopausal patient with a primary carcinoid tumor arising within a mature cystic ovarian teratoma. The carcinoid tumor exhibited a rare combination of histopathological patterns, specifically trabecular and mucinous forms. Furthermore, the patient did not exhibit symptoms of carcinoid syndrome.

Notably, this case is remarkable due to its rarity and the presence of a histopathological pattern that deviates from the typical presentation of primary ovarian carcinoids.

## Case presentation

A 67-year-old multiparous woman sought medical attention for nonspecific lower abdominal pain persisting for two months. The pain was not associated with weight loss, skin flushing, diarrhea, or postural hypotension. However, the patient reported intermittent constipation and urinary incontinence. She was a smoker, and her obstetric history included eight spontaneous vaginal deliveries and bilateral tubal ligation. Menopause occurred at 49 years of age.

**Figure 1:**
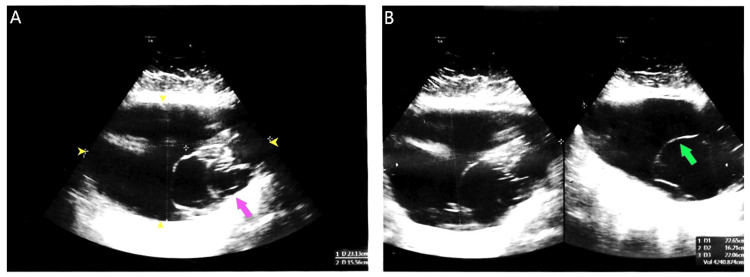
Ultrasonography of the abdomen and pelvis showing A) 23.1x15.5x22.0 cm complex multiloculated cystic lesion (yellow arrowheads) with a thick outer wall (purple arrow) and B) multiple internal septations (green arrow).

On examination, the patient exhibited tenderness in the left lower quadrant of the abdomen. She had an elevated body mass index of 39 kg/m², classifying her as obese. Ultrasonography of the abdomen and pelvis revealed a large, complex, multiloculated cystic lesion measuring 23.1×15.5×22.0 cm. The lesion originated in the pelvis and extended to the epigastrium (Figure 1A and Figure 1B).

Multiple internal septations were noted within the cyst, with no solid components detected. The ovaries were not separately identifiable, and the findings suggested a primary ovarian neoplasm. Other laboratory assessments, including CA-125 levels (18.8 U/mL; reference range: <35 U/mL), were unremarkable.

Subsequent laparoscopy revealed a large cystic mass arising from the left ovary, which was inseparable. The cyst was carefully aspirated and excised, along with the left ovary, for histopathological examination. The patient’s postoperative recovery was uneventful, and she was discharged on the second postoperative day.

**Figure 2:**
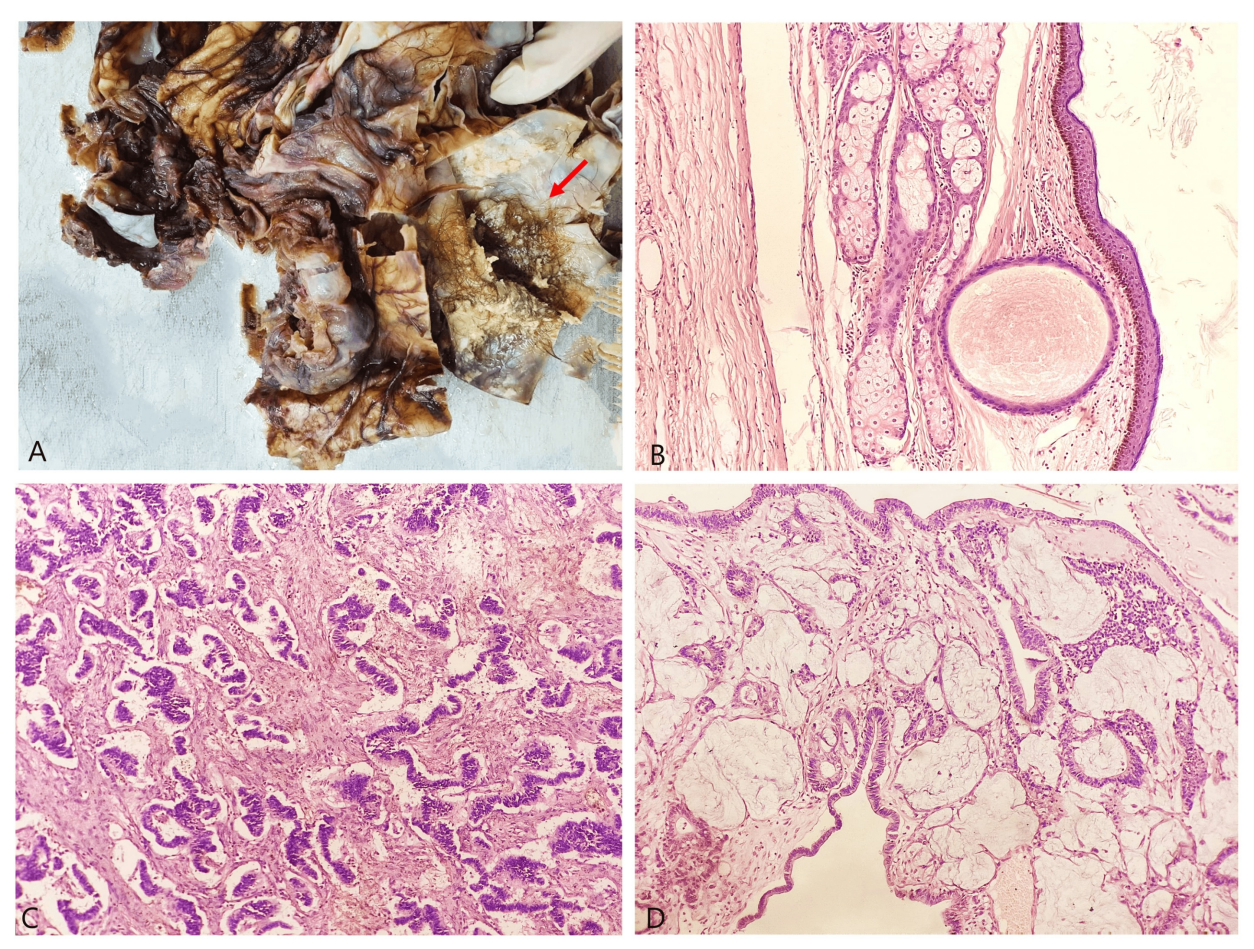
A) Gross section of the specimen showing multiloculations with a large locule containing cheesy material and hair (arrow); B) histopathological examination with hematoxylin and eosin stain of the largest locule showing mature cystic teratoma (dermoid cyst) lined by skin and adnexa, original magnification ×40; C) sections from tan-white fleshy solid areas displaying a carcinoid tumor with cell arrangement in both pseudoacinar and trabecular patterns on hematoxylin and eosin stain, original magnification ×200; D) extracellular mucinous pool with floating islands of carcinoid cells and small round glands lined by cuboidal epithelium as seen with hematoxylin and eosin stain, original magnification ×40.

A gross examination of the excised cyst, measuring 20×18.5×15 cm, revealed an outer smooth capsular surface with no observable deposits or capsule rupture. Serial sectioning displayed multiloculations, including a large locule containing cheesy material and hair (Figure 2A). Adjacent to this locule, numerous small locules filled with mucinous fluid were identified. Additionally, a small 1.8×1.4×1.2 cm tan-white fleshy solid area was present between the mucinous locules and adjacent to the largest locule.

Histopathological examination confirmed the largest locule as a mature cystic teratoma (dermoid cyst), characterized by the presence of skin and adnexal structures (Figure 2B). No immature components were identified. The mucinous locules were lined by mucinous columnar epithelium, displaying histological patterns ranging from a bland single layer to regions with cellular stratification. No significant nuclear atypia or mitotic activity was observed.

**Figure 3:**
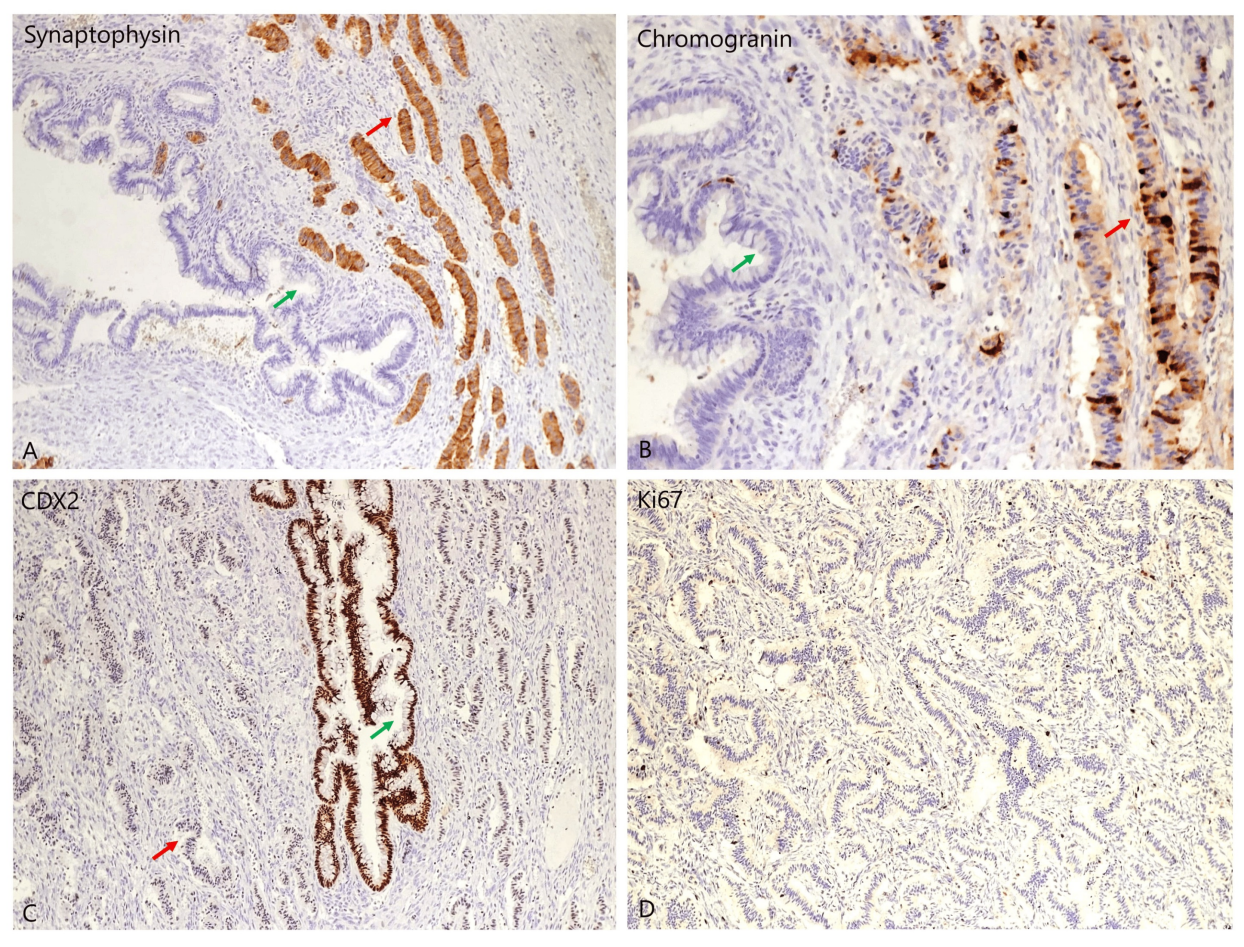
A,B: Synaptophysin and chromogranin immunohistochemical markers showing diffuse staining in the cells of carcinoid component (red arrow) and are negative in the areas of teratoma component (green arrow), original magnification A: x40, B: x100; C: The CDX2 immunohistochemical marker showing positive result for the mucinous component of teratoma (green arrow), and consistently negative result for the trabecular component of carcinoid (red arrow), original magnification x40; D: The Ki67 index is less than 1% favoring the diagnosis of a primary carcinoid tumor, original magnification x200.

Sections from the tan-white fleshy solid area revealed a carcinoid tumor, exhibiting pseudoacinar and trabecular patterns (Figure 2C). These cells featured uniform, small, rounded nuclei with fine granular chromatin. Areas of extracellular mucinous pooling were noted, with floating islands of carcinoid cells and small round glands lined by columnar epithelium (Figure 2D). Immunohistochemical staining with synaptophysin and chromogranin demonstrated diffuse positivity in the typical carcinoid tumor cells (Figures 3A, 3B). The CDX2 marker was positive in the mucinous component but negative in the trabecular component (Figure 3C). Furthermore, the Ki67 index was <1%, indicating a primary origin of the carcinoid tumor rather than metastasis (Figure 3D).

A diagnosis of unilateral mature ovarian teratoma with a localized primary carcinoid tumor was established. The patient underwent regular monitoring with abdominal and pelvic ultrasonography at three-month intervals. A follow-up examination at 12 months revealed no signs or symptoms of tumor recurrence. Written informed consent was obtained from the patient prior to the publication of this case report.

## Discussion

Ovarian carcinoids are rare tumors of the gynecologic tract and exhibit diverse clinical presentations. When carcinoid tumors originate within a teratoma, they may represent a malignant transformation and are believed to arise from neuroendocrine cells present in the gastrointestinal or respiratory epithelium. Pure or primary ovarian carcinoids are categorized as monodermal teratomas and are thought to develop independently [7]. The morphological variations of these tumors include insular, strumal, trabecular, mucinous, and mixed types [8]. The term "strumal carcinoid" is used when an ovarian carcinoid arises in the presence of struma ovarii, which involves thyroid tissue in the ovary.

Among the various subtypes, insular tumors are more likely to induce carcinoid syndrome, as vasoactive amines can enter the systemic circulation through the ovarian veins, bypassing hepatic metabolism [9]. Notably, our case did not exhibit signs of carcinoid syndrome. Furthermore, trabecular carcinoid tumors are known to produce peptide YY, a hormone that reduces intestinal motility and causes constipation [10].

Detecting early malignant transformations during the gross inspection of mature cystic teratomas can be challenging. Therefore, an accurate diagnosis often necessitates thorough microscopic examination and immunohistochemistry [11]. In our case, a gross examination of the teratoma revealed atypical mucinous cystic areas and solid fleshy nodules, raising suspicion of malignant transformation. However, no atypical features or aggressive histopathological characteristics were observed.

The presence of a mucinous carcinoid should prompt consideration of the differential diagnosis, including metastasis from the gastrointestinal tract, particularly the mucinous carcinoids of the appendix. Baker et al. reported that three of six cases of well-differentiated mucinous carcinoids were associated with a mature cystic teratoma [12]. Distinguishing between primary and metastatic disease based solely on morphological characteristics can be challenging. However, this distinction is crucial for prognostic and clinical purposes.

Certain factors may help differentiate primary from metastatic disease. These include smaller tumor size (less than 3 cm), unilateral involvement, absence of surface involvement, and presence of teratomatous components, all of which suggest a primary carcinoid tumor. Conversely, bilateral ovarian involvement, lymphovascular invasion, a multinodular growth pattern, and the absence of teratomatous components favor metastasis [12,13]. In our case, the tumor measured 1.8 cm, exhibited unilateral involvement without surface invasion, and was associated with a dermoid cyst, all indicative of a primary carcinoid tumor. 

It is worth noting that no specific immunohistochemical marker definitively distinguishes primary from metastatic disease. Primary ovarian carcinoids typically exhibit a low Ki67 proliferation index, usually less than 1%, indicating a high level of differentiation [14]. Zhang et al. compared Ki67 indices between primary (median 2.3%, range 0.6%-8.4%) and metastatic carcinoid tumors (median 9.7%, range 1.3%-46.7%) [15]. In our case, the Ki67 index was <1%, supporting the diagnosis of a primary carcinoid tumor (Figure 3D). However, no consensus exists regarding the grading of neuroendocrine tumors in the ovary.

The prognosis of primary ovarian carcinoids is generally favorable, as most subtypes exhibit a benign course with low malignant potential. However, mucinous carcinoids are considered more aggressive, with the potential to spread beyond the ovaries [16].

Currently, no specific guidelines exist for managing primary ovarian carcinoid tumors, and treatment options vary depending on the extent of the disease and the patient’s age. In premenopausal women with unilateral disease, fertility-preserving surgery, such as unilateral oophorectomy, is typically preferred. In postmenopausal women or cases involving mucinous carcinoid tumors, more radical options, such as total abdominal hysterectomy with bilateral oophorectomy, may be recommended. In our case, we performed an oophorectomy of the affected side only, as intraoperative findings did not indicate bilateral involvement or advanced disease.

Finally, there are no consensus guidelines in the literature regarding follow-up management of primary ovarian carcinoids with mucinous components, as their clinical behavior remains poorly defined. For this patient, we established a specific follow-up plan. Close postoperative monitoring with abdominal and pelvic ultrasonography every three months is recommended, followed by CT scans of the abdomen and pelvis at one-year intervals and a PET scan at three-year intervals. In the event of recurring symptoms, immediate CT or PET scanning should be performed to rule out malignancy recurrence.

## Conclusions

This case highlights the rare occurrence of a mixed trabecular and mucinous carcinoid tumor arising within a mature cystic ovarian teratoma in a postmenopausal woman. Despite the absence of carcinoid syndrome, the diagnosis was achieved through thorough histopathological and immunohistochemical evaluation, emphasizing the importance of detailed postoperative assessment for identifying rare tumor subtypes. The presence of a mucinous component underscores the need for vigilant postoperative monitoring to rule out metastatic origins and potential recurrence. This report contributes to the limited literature on primary ovarian carcinoids and supports the importance of individualized follow-up strategies in such cases.
